# Transforming Growth Factor Beta 2 and Heme Oxygenase 1 Genes Are Risk Factors for the Cerebral Malaria Syndrome in Angolan Children

**DOI:** 10.1371/journal.pone.0011141

**Published:** 2010-06-16

**Authors:** Maria Rosário Sambo, Maria Jesus Trovoada, Carla Benchimol, Vatúsia Quinhentos, Lígia Gonçalves, Rute Velosa, Maria Isabel Marques, Nuno Sepúlveda, Taane G. Clark, Stefan Mustafa, Oswald Wagner, António Coutinho, Carlos Penha-Gonçalves

**Affiliations:** 1 Instituto Gulbenkian de Ciência, Oeiras, Portugal; 2 Hospital Pediátrico David Bernardino, Luanda, Angola; 3 Faculdade de Medicina, Universidade Agostinho Neto, Luanda, Angola; 4 Departments of Epidemiology and Public Health and Infectious and Tropical Diseases, London School of Hygiene and Tropical Medicine, London, United Kingdom; 5 Department of Laboratory Medicine, Medical University of Vienna, Vienna, Austria; Federal University of São Paulo, Brazil

## Abstract

**Background:**

Cerebral malaria (CM) represents a severe outcome of the *Plasmodium falciparum* infection. Recent genetic studies have correlated human genes with severe malaria susceptibility, but there is little data on genetic variants that increase the risk of developing specific malaria clinical complications. Nevertheless, susceptibility to experimental CM in the mouse has been linked to host genes including *Transforming Growth Factor Beta 2 (TGFB2)* and *Heme oxygenase-1 (HMOX1)*. Here, we tested whether those genes were governing the risk of progressing to CM in patients with severe malaria syndromes.

**Methodology/Principal Findings:**

We report that the clinical outcome of *P. falciparum* infection in a cohort of Angolan children (n = 430) correlated with nine *TGFB2* SNPs that modify the risk of progression to CM as compared to other severe forms of malaria. This genetic effect was explained by two haplotypes harboring the CM-associated SNPs (Pcorrec. = 0.035 and 0.036). In addition, one *HMOX1* haplotype composed of five CM-associated SNPs increased the risk of developing the CM syndrome (Pcorrec. = 0.002) and was under-transmitted to children with uncomplicated malaria (P = 0.036). Notably, the *HMOX1*-associated haplotype conferred increased *HMOX1* mRNA expression in peripheral blood cells of CM patients (P = 0.012).

**Conclusions/Significance:**

These results represent the first report on CM genetic risk factors in Angolan children and suggest the novel hypothesis that genetic variants of the *TGFB2* and *HMOX1* genes may contribute to confer a specific risk of developing the CM syndrome in patients with severe *P. falciparum* malaria. This work may provide motivation for future studies aiming to replicate our findings in larger populations and to confirm a role for these genes in determining the clinical course of malaria.

## Introduction

Cerebral malaria (CM) is an acute life threatening complication of clinical malaria that afflicts a fraction of the *P. falciparum*-infected patients and it is perceived as a complex syndrome, rather than an isolated entity [1], [2], [3]. While potentially reversible, CM can develop into an unfettered neurovascular inflammatory syndrome leading to irreversible coma [3], [4]. Studies on malaria heritability indicate that about 25% of the risk to progress to severe malaria is determined by human genetic factors [5], [6]. The strong protection against severe malaria conferred by the *HbS* [7], G6PD [8], [9], [10] and the alpha-thalassemia mutations [11], [12], [13] illustrate the striking selection pressure of malaria on the human genome.

Genetic variation may in part determine the epidemiological patterns of susceptibility/resistance to malaria observed at the population level, whereby a fraction of the individuals infected with the malaria parasite would present parasitaemia and uncomplicated disease, a fraction of uncomplicated disease cases would progress to severe disease and a fraction of severe disease cases would develop CM syndrome [14]. Presumably, the genetic factors controlling malaria severity would affect the risk of disease progression from uncomplicated malaria, characterized by low levels of parasitaemia and fever, towards the different severe clinical complications and outcomes. Although many studies emphasize the role of genetic factors in controlling the severity of the malaria infection, in most instances the study design does not allow comparisons of CM with non-cerebral forms of severe malaria [15], [16], [17], [18] precluding the identification of genetic variants that specify the risk of developing CM.

We performed a genetic study to test the prior hypothesis that host factors influence specifically the risk of developing CM syndrome in the context of severe malaria. The study design aimed to identify candidate genetic variants associated to CM by comparing a group of CM patients with a group of severe malaria patients that did not exhibit CM. This hospital-based study was carried out in Luanda, a province of Angola that represents a mesoendemic stable region and where malaria is the first cause of morbidity and mortality [19]. Malaria transmission in Angola is perennial with enhancement during the rainy season and *P. falciparum* malaria is the most frequent infection (Angolan Ministry of Health, MINSA 2001).

The study of CM pathogenesis has benefited from investigations on experimental systems that use artificial laboratory infections to develop rodent models of experimental cerebral malaria (ECM). This report focused on four functional candidate genes (*TGFB2*, *HMOX1*, *ICAM1 and CD36*) that have been correlated with CM and other forms of severe malaria in mouse models. To our knowledge the *TGFB2* gene has not been studied in the context of human malaria but it was suggested to confer resistance to ECM in a wild mouse strain. Heme oxygenase -1, encoded by *Hmox1* gene, was shown to prevent the development of ECM but its role in human CM has not been addressed other than by an exploratory study in CM patients in Myanmar [20]. The *ICAM 1* and *CD36* genes have also been related to ECM in the mouse models [21], [22] but investigations on the role of these genes in human CM and severe malaria led to inconclusive and often contradictory results [15], [23], [24], [25], [26], [27], [28], [29].

This work represents an initial report on CM genetics in the Angolan population and though requiring further confirmation in larger samples from other populations, the results suggest that *TGFB2* gene variants contribute to the risk of developing the CM syndrome when compared to other forms of severe malaria in Angolan children. The results also suggest that *HMOX1* gene variants are associated to CM risk and control the *HMOX1 m*RNA expression in peripheral blood cells of children with CM.

## Results

The sample population in this study consisted of 430 children diagnosed with malaria in the central pediatric hospital of Luanda (Angola) and the 319 uninfected apparently healthy controls, all attendees to the vaccination ward of the same hospital. A fraction of the CM patients (16.9%) showed severe malaria anemia and 11.5% also had hyperparasitaemia. Nevertheless, we did not find association between hyperparasitaemia and severe anemia (Chi-Square test, P value = 0.58) and 47.7% of the CM patients had low parasitaemia levels, suggesting that high levels of parasitaemia were not a requirement to develop CM. The ethnic group Kimbundu that predominates in Luanda was the most represented in our study (50.7%), while Umbundu was the second ethnic group (13.7%). Nevertheless, the ethnic composition did not differ among patient groups (Kruskal-Wallis Test, P-Value = 0.56) and there was no association between ethnic groups and malaria outcome (Chi-Square test, P-value = 0.74) suggesting that ethnic ancestry was not a major factor in stratifying the clinical malaria phenotypes.

All individuals were genotyped and analyzed for 54 single nucleotide polymorphisms (SNPs) that covered a region of 100Kb that encompassing the *TGFB2* gene (*Transforming growth factor*, *beta 2*), 3.1 Kb in the *CD36* gene (*Thrombospondin receptor*), 3.3 Kb in the *HMOX1* (*Heme oxygenase -1*) *gene region*, 5.2 Kb in the *ICAM 1* (*Intercellular adhesion molecule 1*, *CD54*) gene region as well as dbSNP rs334 which defines the *HbS* mutation. The complete list of SNPs that passed genotyping quality control and were subjected to association analysis is shown in Table S1. For the analyzed SNPs the Hardy-Weinberg (HWE) was preserved in all patient groups and uninfected apparently healthy controls, except for the *HbS* mutation that was in equilibrium in the healthy controls but not in the malaria patient groups.

To identify genetic polymorphisms specifically associated to CM, we used the strategy of comparing genotype frequencies between 130 patients with the CM syndrome and 158 patients with severe forms of malaria severe malaria in absence of CM (SnC). To better define CM-associated genetic factors that are exclusively detected when comparing CM to SnC patients, the study included a group of 142 patients with uncomplicated malaria (UM) and 319 uninfected healthy controls (UIF). The evaluation of the genetic association tests took into account that the sample size limited the resilience to multiple test corrections. Therefore, the initial single marker analysis was used to identify SNPs with suggestive effects on the CM phenotype and the subsequent analysis was pursued in the context of haplotype association using corrected P values (see methods section).

### Single marker analysis

Genotype frequency analysis showed the frequency of the *HbS* mutation (rs334) did not differ significantly between CM and SnC patients but was notably higher in UM patients and UIF controls (Table 1). These results confirmed that the well-documented protective effect of the *HbS* mutation against severe malaria [7], [30] is measurable in the Angolan population and suggested that the sickle cell anemia trait is not specifically protecting from CM development.

**Table 1:** Association results for cerebral malaria risk single-nucleotide polymorphisms (SNPs) genotyped in *TGFB2*, *CD36*, *HBB* and *HMOX1* genes.

GENE	dbSNP (RA)	RA frequency (%)				P Odds Ratio (CI)		
		CM (2n = 260)	SnC (2n = 316)	UM (2n = 284)	UIF (2n = 638)	CM/SnC	CM/UM	CM/UIF
TGFB2	rs1317681(T)	17.5	12.3	13.1	15.3	0.0471.72 (1.00–2.96)	1.52 (0.88–2.60)	1.27 (0.81–1.99)
	rs1934852(A)	56.6	46.7	51.8	50.6	0.0341.79 (1.04–3.12)	1.43 (0.83–2.44)	1.32 (0.83–2.08)
	rs6657275(G)	56.2	47	51.4	50.2	0.041.75 (1.02–2.94)	1.43 (0.85–2.44)	1.35 (0.86–2.08)
	rs1342586(T)	18.9	11.1	15.7	14.7	0.0151.95 (1.14–3.34)	1.28 (0.76–2.15)	1.31 (0.84–2.04)
	rs4846478(G)	43.4	34.6	39.1	38.6	0.0341.70 (1.04–2.79)	1.29 (0.78–2.15)	1.38 (0.89–2.14)
	rs6684205(G)	56.0	47.0	51.4	50.0	0.0461.72 (1.01–2.94)	1.43 (0.83–2.44)	1.33 (0.85–2.08)
	rs1418553(C)	56.0	46.7	51.4	50.0	0.0411.75 (1.02–3.03)	1.43 (0.83–2.43)	1.33 (0.85–2.08)
	rs900(T)	43.0	34.3	38.7	39.1	0.031.70 (1.04–2.77)	1.30 (0.79–2.15)	1.32 (0.85–2.03)
	rs1473527(A)	71.0	64.9	69.1	66.9	0.031.69 (1.05–2.78)	1.02 (0.63–1.64)	1.37 (0.90–2.08)
CD36	rs1194182(G)	40.2	29.9	32.3	31.4	0.0161.51 (1.08–2.12)	1.37 (0.97–1.92)	0.0032.65 (1.46–4.80)
	rs3211821(G)	41.5	31.9	29.9	33.7	0.0241.48 (1.05–2.08)	0.0061.64 (1.14–2.34)	0.0331.39 (1.03–1.87)
	rs1358337(C)	46.3	41.1	36.8	39.9	1.22 (0.88–1.70)	0.0381.42 (1.02–1.98)	0.0251.82 (1.09–3.05)
HBB	rs334(T)	2.7	4.6	11.0	15.1	0.58 (0.19–1.73)	0.00090.22 (0.08–0.60)	0.1×10^−9^0.11 (0.04–0.28)
HMOX1	rs2071748(A)	70.9	71.8	62.8	65.3	1.05 (0.72–1.53)	0.0431.47 (1.01–2.12)	1.28 (0.93–1.75)
	rs8139532(G)	55.2	48.3	52.5	53.0	0.0251.85 (1.07–3.22)	1.35 (0.8–2.33)	1.27 (0.81–2.0)
	rs7285877(C)	55.3	48.0	53.6	53.4	0.0241.85 (1.07–3.22)	1.25 (0.74–2.13)	1.27 (0.80–2.0)
	rs11912889(G)	54.4	47.3	50.7	52.8	0.0321.81 (1.05–3.22)	1.30 (0.76–2.22)	1.31 (0.83–2.08)

Abbreviations: CM, cerebral malaria; SnC, severe no cerebral malaria; UM, uncomplicated malaria; UIF, uninfected; RA, reference allele. P-values and Odds Ratios were obtained by logistic regression analysis. Allele frequencies refer to the reference allele conferring susceptibility or resistance to CM.

To identify genetic polymorphisms that specifically modify the risk of developing CM, we searched for genotype differences between patients having CM and those having severe malaria in absence of CM (SnC). We found that the genotype frequencies in 9 out of the 26 SNPs analyzed in the *TGFB2* gene and 3 out of 10 SNPs analyzed in *HMOX1* gene showed suggestive association to CM (uncorrected P-value<0.05) when comparing CM patients with SnC patients but not in comparison with UM patients and UIF controls (Table 1). These results raised the possibility that variants of these genes could be involved in specifying the risk of developing CM in the context of severe malaria. Also, we noted that an additional SNP in the *HMOX1* gene (rs2071748) showed significant association only when comparing CM with UM patients (Table 1). This finding suggested that the SNP rs2071748 could represent a risk factor for severe malaria, which was also detected when comparing SnC to UM patients (data not shown). This SNP maps very closely to the GT repeat polymorphism that has been associated to CM in a study in the Myanmar population [41]. We genotyped this microsatellite and found that the frequency of the GT-repeat short alleles tends to be higher in CM patients as compared to UIF controls (P = 0.0057) and to UM patients (P = 0.038) but not when compared to SnC patients (see Figure S1 and Table S2). This result suggests that the rs2071748 CM-association signal was also captured by the HMOX1-GT repeat. In contrast, two SNPs on the *CD36* gene which was previously reported to be associated with disease severity [28], [31], [32], showed to be associated to CM when comparing to SnC patients but also with UM patients and UIF controls (Table 1). This result suggested that genetic variants of the *CD36* gene play a role at various stages of disease progression but are not specifically involved in specifying the clinical outcome of the severe malaria. We also found that the SNPs of the *ICAM 1* gene were not associated to CM (Table S3).

### Linkage Disequilibrium analysis

**Figure 1:**
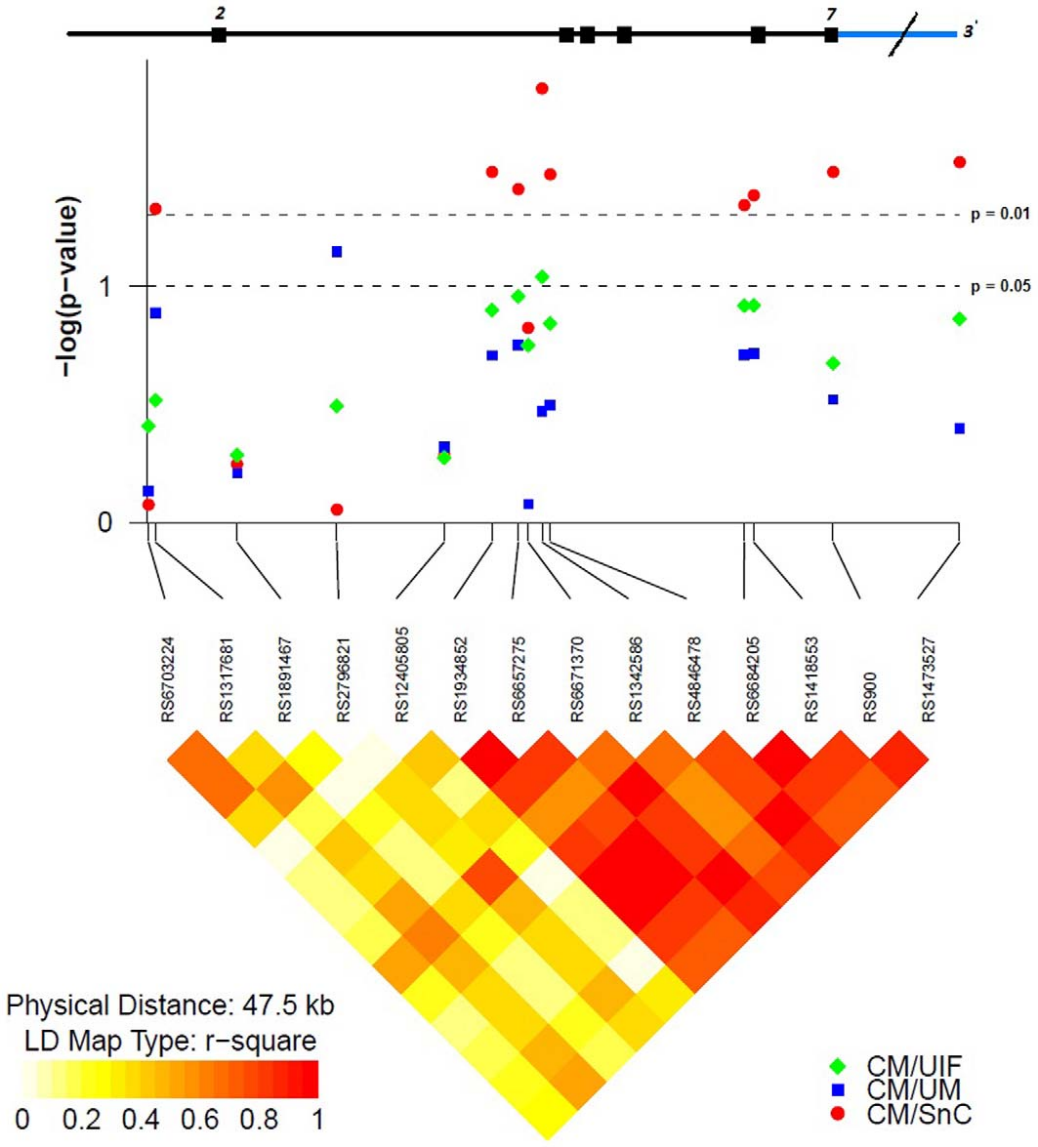
Association tests and LD analysis in the *TGFB2* gene single-nucleotide polymorphisms (SNPs). The diagram represents in the vertical axis, the log10 of the P value of association tests of SNPs listed in the y-axis, obtained from logistic regression analysis. The points in red, blue and green represent the results of the association with the SNPs between cases of cerebral malaria (CM) and controls with severe no cerebral malaria (SnC), uncomplicated malaria (UM) and uninfected (UIF), respectively. The upper diagram shows a scale representation of the structure of the *TGFB2* gene: exons are represented by boxes and marked with its number. The bottom of the diagram represents the LD between a pair of SNPs measured by r^2^. The image, below and to the left, corresponds to the color scale representing the values of LD, from yellow (r^2^ = 0) to red (r^2^ = 1). This diagram is an adaptation of the figure produced by the snp. plotter R package.

**Figure 2:**
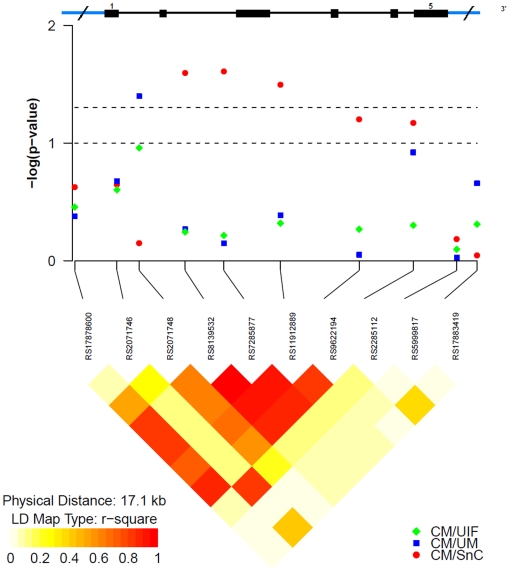
Association tests and LD analysis in the *HMOX1* gene single-nucleotide polymorphisms (SNPs). The diagram represents in the vertical axis, the log10 of the P value of association tests of SNPs listed in the y-axis, obtained from logistic regression analysis. The points in red, blue and green represent the results of the association with the SNPs between cases of cerebral malaria (CM) and controls with severe no cerebral malaria (SnC), uncomplicated malaria (UM) and uninfected (UIF), respectively. The upper diagram shows a scale representation of the structure of the *HMOX1* gene: exons are represented by boxes and marked with its number. The bottom of the diagram represents the LD between a pair of SNPs measured by r^2^. The image, below and to the left, corresponds to the color scale representing the values of LD, from yellow (r^2^ = 0) to red (r^2^ = 1). This diagram is an adaptation of the figure produced by the snp. plotter R package.

We used linkage disequilibrium (LD) analysis to take into account the relative positions of the CM-associated SNPs in the *TGFB2* and *HMOX1* genes. Out of the 26 SNPs that were analyzed across 100 kb in the *TGFB2* region, the nine SNPs specifically associated with CM clustered in a region spanning 48 Kb. LD analysis revealed a region of strong LD covering 27 kb of the structural *TGFB2* gene that encompassed eight SNPs associated with CM. The remaining CM-associated SNP (rs1317681) maps 15 kb from this region and showed a weaker statistical association to CM (Table 1). The r^2^ among the eight SNPs varied from 0.6 to 1.0 suggesting that this region may represent a LD block in the *TGFB2* gene (Figure 1). Similarly, we found that 5 out the 10 SNPs analyzed in the *HMOX1* gene were mapping within a strong LD group (r^2^>0.9) that spanned approximately 8 kb. This region encompassed the three SNPs associated with CM, one SNP (rs9622194) marginally associated with CM (P-value = 0.06) (Table S3) and the SNP (rs2071748) associated with disease severity (Figure 2).

### Haplotype association

The results of the LD analysis were used to reconstruct haplotypes (R package GAP) of the *TGFB2* and *HMOX1* genes and test whether the most frequent haplotypes (>5%) were associated to CM correcting statistical significance using permutation tests. Using the eight SNPs that showed exclusive CM-specific association within the *TGFB2* LD block we reconstructed six haplotypes that were denoted TGFB2a to TGFB2e (Table 2). The analysis confirmed the overall association (Global Score Statistics) of the *TGFB2* haplotypes when comparing CM with SnC patients (corrected P-value = 0.041). TGFB2c and TGFB2f haplotypes with distinct alleles in all seven CM-associated SNPs, showed opposite effects on the risk of developing CM (Table 2). The TGFB2c was significantly more frequent in CM patients than in those with other forms of severe malaria (corrected P-value = 0.035) and its genetic effect appears to be restricted to increase the risk of developing CM in the context of severe malaria. In contrast, the TGFB2f haplotype that was less frequent in the population significantly decreased the risk of progression to CM (corrected P value = 0.036). This protective effect is inferred from the reduction of the TGFB2f haplotype frequency among the CM patients that is still detectable when comparing CM patients to UIF controls (Table 2).

**Table 2:** Association results for *TGFB2* haplotypes.

Haplotypes	Haplotype frequencies (%)				Cerebral Malaria *versus* Controls P (Hap-Score)		
	CM (2n = 260)	SnC (2n = 316)	UM (2n = 284)	UIF (2n = 638)	CM/SnC^a^	CM/UM^b^	CM/UIF^c^
TGFB2aCAGCCACAG	29.0	35.8	32.5	32.9	0.83(−1.68)	0.40(−0.79)	0.28(−1.07)
TGFB2bAGACGGTTA	24.0	23.0	23.5	23.6	0.80(0.27)	0.90(0.11)	0.93(0.06)
TGFB2cAGATGGTTA	18.3	11.5	16.0	14.8	0.035**(2.21)**	0.33(0.96)	0.20(1.36)
TGFB2dAGATCGTAA	13.7	12.5	11.2	11.7	0.70(0.41)	0.60(0.69)	0.57(0.65)
TGFB2eCAGCCACAA	9.8	7.6	7.5	8.5	0.36(0.89)	0.34(0.86)	0.47(0.83)
TGFB2fCAACCACAA	4.7	9.7	9.3	8.3	0.036**(−2.26)**	0.037**(−2.08)**	0.049**(−1.92)**

Abbreviations: CM, cerebral malaria; SnC, severe no cerebral malaria; UM, uncomplicated malaria; UIF, uninfected. Hap-Score represents susceptibility (positive values) or resistance (negative values). In each haplotype are sequentially represented the alleles from SNPs of the LD block: rs1934852, rs6657275, rs6671370, rs1342586, rs4846478, rs6684205, rs1418553, rs900 and rs1473527. Haplotypes represented have frequency >5% in UIF. Global Score Statistics Corrected P: a) 0.041; b) 0.26; c) 0.41.

Haplotype reconstruction using the five SNPs in the LD block of the *HOMX1* gene identified three haplotypes denoted HMOX1a, HMOX1b and HMOX1c (Table 3). Association analysis revealed that the HMOX1c haplotype frequency was significantly higher in CM patients as compared to SnC and UM patients as well as to uninfected controls (Table 3). These results suggest that the HMOX1c haplotype has a general role as a CM syndrome susceptibility factor particularly when compared to uncomplicated malaria patients (P = 0.002). On the other hand, the HMOX1b haplotype showed a CM protective effect with marginal significance when comparing CM with UM patients (corrected P-value = 0.056). In fact, the frequency of the HMOX1b haplotype was similar in CM patients and in patients with severe non-cerebral malaria. This finding suggested that this haplotype act at different stages of infection as a factor conferring protection from severe malaria (Table 3).

**Table 3:** Association results for *HMOX1* haplotypes.

Haplotypes	Haplotype frequencies (%)				Cerebral Malaria *versus* Controls P (Hap-Score)		
	CM (2n = 260)	SnC (2n = 316)	UM (2n = 284)	UIF (2n = 638)	CM/SnC^a^	CM/UM^b^	CM/UIF^c^
HMOX1aAATAA	41.3	45.7	42.1	43.2	0.37(−0.90)	0.94(−0.04)	0.61(−0.50)
HMOX1bGGCGG	29.1	28.0	37.5	34.7	0.70(0.36)	0.06**(−1.94)**	0.22(−1.26)
HMOX1cAGCGG	22.7	14.8	11.7	14.6	0.03**(2.12)**	0.002**(3.06)**	0.01**(2.41)**

Abbreviations: CM, cerebral malaria; SnC, severe no cerebral malaria; UM, uncomplicated malaria; UIF, uninfected. Hap-Score represents susceptibility (positive values) or resistance (negative values). In each haplotype are sequentially represented the alleles from SNPs of the LD block: rs2071748, rs8139532, rs7285877, rs11912889 and rs9622194. a) Global Score Statistics Corrected P = 0.06; b) P = 0.009; c) P = 0.10.

### Transmission disequilibrium tests

We used transmission disequilibrium tests (TDT) in mother-affected child pairs to corroborate the haplotype associations observed in the case-control analysis. The TDT analysis was performed with the TRANSMIT software using 105 mother-affected child pairs with CM, 115 with SnC and 106 with UM. The TDT analysis detected the protective role of the *HbS* mutation as it was under-transmitted to CM children (P = 0.036) (Table 4). Moreover, we found that the TGFB2c haplotype (AGATGGTTA), deemed to be overrepresented in severe malaria patients that progressed to CM, was under-transmitted to SnC children that did not progress to cerebral malaria (P = 0.038) (Table 4). On the other hand, the HMOX1c haplotype (AGCGG) was found to be under-transmitted to UM children (P = 0.036), reinforcing the hypothesis that this haplotype represents a risk factor for severe malaria (Table 4). Overall, the TDT results corroborated the initial case-control association findings for haplotypes that showed relatively high frequency. Failure to confirm associations of less frequent haplotypes (e.g. TFB2f) by TDT is presumably due to reduced statistical power. Nevertheless, the results of the TDT analysis suggested that the associations of *TGFB2* and *HMOX1* genes to CM are not attributable to population stratification effects in groups of patients and controls.

**Table 4:** Transmission disequilibrium tests for associated *TGFB2* and *HMOX1* haplotypes and dbSNP rs334.

Haplotype/dbSNP	Phenotype	Observ.	Expec.	Var (O-E)	P
TGFB2cAGATGGTTA	CM	20.0	18.9	5.3	0.65
	SnC	7.3	11.1	3.2	0.038
	UM	13.9	12.6	3.9	0.53
TGFB2fCAACCACAA	CM	8.0	7.9	2.6	0.92
	SnC	5.7	5.1	1.7	*
	UM	5.0	3.7	1.1	*
HMOX1bGGCGG	CM	56.3	59.4	15.2	0.49
	SnC	67.8	69.1	14.7	0.72
	UM	75.9	71.5	14.7	0.30
HMOX1cAGCGG	CM	42.2	39.8	10.8	0.54
	SnC	34.2	37.0	10.5	0.42
	UM	23.1	32.5	9.0	0.0036
rs334	CM	7.0	11.4	3.8	0.036

*Low frequency in the mother-descendent pairs precluded the analysis. Abbreviations: CM, cerebral malaria; SnC, severe no cerebral; Var (O-E), variance (observed-expected).

### *HMOX1* SNPs control *HMOX1* mRNA expression in children developing CM

To investigate the functional basis of *HMOX1* association to malaria we examined whether the *HMOX1* haplotypes associated to the clinical outcomes of malaria were also controlling the level of *HMOX1* gene expression among children developing CM. Relative expression of *HMOX1* mRNA in 42 children developing CM was quantified by Real-Time PCR in peripheral blood leukocytes. The basal levels were fairly high, as judged from the Real-Time PCR CTs with prominent curves. Quantitative trait locus (QTL) analysis detected suggestive association of the HMOX1c haplotype (AGCGG) to the level of *HMOX1* mRNA expression (P≤0.01) (Table 5). This result revealed that the HMOX1c genetic variant that conferred increased risk to develop CM is also associated to higher expression of *HMOX1* mRNA in the context of CM.

**Table 5:** Haplotypic quantitative trait analysis of *HMOX1* gene expression in cerebral malaria patients.

Haplotype	BETA	STAT	P
HMOX1aAATAA	−1.88	3.82	0.06
HMOX1bGGCGG	0.36	0.12	0.74
HMOX1cAGCGG	3.18	8.70	0.012

Beta, genetic effect under logistic regression model; Stat, test statistic; P, empirical p-value.

## Discussion

In this work, we aimed to identify genetic variants specifying the emergence of CM in severe malaria patients by contrasting genotype frequencies in those patients presenting CM with those exhibiting signs of severe malaria but exempt of CM symptoms. Single marker analysis suggested that SNPs in the *TGFB2* and *HMOX1* genes showed specific association to the CM syndrome. In addition, haplotype association analysis showed that the TGFB2c haplotype specifically increased the risk of developing CM as compared to other severe malaria complications. This haplotype was also under-transmitted to patients with severe non-cerebral malaria. On the other hand, the HMOX1c haplotype seemed to play a general role in CM risk as it was overrepresented in CM patients as compared to SnC and UM patients as well as to uninfected controls. In accordance, this haplotype was under-transmitted to patients with uncomplicated malaria. Moreover, QTL analysis showed that the HMOX1c haplotype was associated to higher expression of *HMOX1* mRNA in peripheral blood cells of CM patients.

We did not find that *CD36* was a determinant of the risk of developing CM as compared to other severe malaria complications. In fact, the role of the *CD36* gene in malaria is still debated and raises the question of whether different studies may capture different effects of the *CD36* gene variants in severe and in cerebral malaria. We found that the SNP rs3211958 was not associated to CM although this SNP is known to be in complete LD with rs3211938 which showed contradictory results in different African populations [15], [26], [28], [33]. In our sample, the absence of CM-association with the *ICAM 1* SNPs is in agreement with the results reported by a study in Gambia, Malawi and Kenya populations [26], though it has been reported that the heme oxygenase-1 has a down-regulating effect on the *ICAM-1* expression levels [34], [35]. Such heterogeneity effects should be kept in mind when judging lack of replication of many reported SNP associations to disparate malaria clinical manifestations [36], [37], [38].

Though the pathogenesis and the symptoms of cerebral malaria in murine CM do not completely overlap the observations in human CM, the mouse models have brought critical insights to our understanding of CM pathogenesis [39], [40], [41]. Thus the *TGFB2* gene was identified as a candidate gene to CM susceptibility in a genetic cross that used wild-derived mouse strains and where CM susceptibility segregated from hyperparasitaemia [42], suggesting that *TGFB2* would be a gene conferring risk to CM in the context of severe malaria. *Tgfb2*−/− mice are not viable and studies with heterozygous mice in the context of malaria infection are warranted and may provide further evidence of involvement of the *Tgfb2* gene in ECM pathogenesis.

Members of the TGF-beta cytokine family have been associated to the control of malaria infection and parasite growth [43], [44], [45] and TGFB isoforms were shown to accumulate in the brain of cerebral malaria patients [46]. The study of the *TGFB2* haplotypic structure in the Angolan population led us to identify an LD block covering 27kb in the structural region of the gene. Although the CM-associated region in the *TGFB2* gene covers part of the coding region, all the SNPs that we analyzed were intronic and have no predicted effects in folding, activity or function of the *TGFB2* protein. Re-sequencing of relevant variants of the *TGFB2* gene may provide further information on putative functional polymorphisms explanatory of the role of *TGFB2* in CM pathogenesis. Studies of *TGFB2* haplotypes in other populations and areas with different malaria endemicity would be required to determine whether the *TGFB2* genetic variance represents a widespread CM risk factor. It should be noted that according to our observations the replication of *TGFB2* specific association to CM in other populations is expected to be stronger when analyzed in the context of severe malaria.

The Heme oxygenase-1 activity has been shown to suppress ECM in mice [47] and one report in Myanmarese patients suggested that short alleles of a GT repeat in the *HMOX1* promotor region are a risk factor for cerebral malaria [20]. Here, we show that one haplotype (HMOX1c) composed by SNPs covering a 8Kb LD block in the structural region of the *HMOX1* gene confers susceptibility to CM which (Table 3). Strikingly, the occurrence of the allele A at position rs2071748 in the context of this haplotype generates the HMOX1b haplotype that marginally decreases the risk to progress from UM to CM. It is worth noting that the A at position rs2071748 is not able to mediate CM susceptibility in the context of the high frequency HMOX1a haplotype (Table 3). We also noted that the rs2071748 SNP was mapping in the *HMOX1* promotor region very closely to the GT repeat polymorphism that showed that the short alleles were overrepresented in our CM patients and in Myanmar patients [20]. We also found that the HMOX1c haplotype governs the expression of *HMOX1* mRNA in peripheral blood leucocytes (PBL) of CM patients. We speculate that *HMOX1* gene expression measured in PBLs could reflect the ability of blood cells to express this gene in the brain. Thus, our results raise the possibility that *HMOX1* variants may control the risk of CM through regulation of *HMOX1* expression. Nevertheless, we cannot exclude that relative differences in PBLs lineages among CM patients could influence *HMOX1* gene expression measurements. Overall, the *HMOX1* association analysis suggests that the involvement of this gene in determining the outcome of malaria infection might be related to disease severity and not exclusively to the CM syndrome.

Our results highlight the value of a study design centered in a particular clinical manifestation of malaria, namely CM, and comparing that clinical manifestation with other frequent outcomes, including non-cerebral forms of malaria (SnC group) or uncomplicated malaria (UM group). This enabled the identification of genetic variants associated with CM in the context of severe malaria syndromes. Thus, the clinical stratification approach we used in this study could prove a useful tool to complement genome-wide association analysis of malaria [48], [49] allowing fine-resolution association analysis of candidate genes and taking into account the haplotypic context and the genetic variability within African populations [50], [51].

This work represents an initial study on genetics of CM in the Angolan population. The statistical evidence supporting the described genetic association findings was limited by the relatively small sample size, by the genetic heterogeneity of the Angolan population, by the presumed small genetic effects and by lack of knowledge of yet to describe causal genetic variants possibly located within the CM associated regions. Although these factors limit the extrapolation of the association results to other populations our findings suggest the novel hypothesis that in African populations the *TGFB2* and *HMOX1* genetic variation correlate with malaria genetic susceptibility.

This report may provide insights and motivation for future association and functional studies aiming to replicate our findings in larger populations and to confirm a role for *TGFB2* and *HMOX1* in determining the course of disease in the response to *Plasmodium* infections.

## Materials and Methods

### Subjects, Phenotypic and Clinical Criteria

A total of 749 children, living in Luanda and ranging from 6 months to 13 years of age were enrolled in the present study. Ethical permission for this study was granted by the Ethical Committee of the Hospital Pediátrico David Bernardino (HPDB) in Luanda that was appointed by the Angolan Ministry of Health. Written, informed consent was obtained from the parents or guardians of each child. Patients were selected among attendance to the HPDB. Uninfected controls were sampled from children randomly selected in the vaccination ward of the HPDB. The sample collection was carried out from February 2005 to May 2007 and comprised 130 CM children (cases), 158 patients with severe malaria but not CM (SnC), 142 patients with uncomplicated malaria (UM) and 319 uninfected controls (UIF). The mean age in months was 54.2 for CM cases, 45.9 for SnC patients, 50.3 for UM patients and 60.9 for UIF controls. Ethnicity was defined on a parental basis and the major ethnic groups were the Kimbundu and Umbundu. Malaria was diagnosed on the basis of a positive asexual parasitaemia detected on a Giemsa-stained thick smear. For parasitaemia quantification the number of parasites per 100 high-power microscopic fields was estimated and the parasite density was calculated from this value [52]. CM was defined according to the WHO criteria: a coma score <3 in Blantyre Scale for children <60 months or a coma score <7 in Glasgow Scale for children ≥60 months. Meningitis and encephalitis were ruled out by cerebrospinal fluid analysis after lumbar puncture. Exclusion criteria were a different known aetiology of encephalopathy and hypoglycaemia (glycaemia <40 mg/dl). A fraction of CM patients showed as part of the CM syndrome additional clinical complications such as severe malaria anaemia (SMA) and hyperparasitaemia (HPM). The severe non-CM group (SnC) included patients with SMA and/or HPM. Children with HPM had ≥100 red blood cells parasitized by one high-power microscopic field and SMA was defined by haemoglobin <5 g/dl or hematocrit <15%. Patients with consciousness disturbances or with other disease were excluded from this group. The uncomplicated malaria (UM) group represents patients with malaria diagnosis and febrile illness without any clinical finding suggestive of other causes of infection and with no manifestations of severe malaria. All the uncomplicated malaria patients were outpatients. Patient treatments followed the established hospital guidelines. Enrollment of uninfected controls excluded children with any clinical finding suggestive of illness and the uninfected status was confirmed by the absence of *Plasmodium* DNA in the peripheral blood as detected by PCR [53].

### Sample Preparation, Candidate Genes and Genotyped SNPs

Genomic DNA was extracted from whole blood using the Chemagen Magnetic Bead Technology. DNA preparations were quantified using PicoGreen reagents according to the supplier instructions.

All the 749 children and their mothers were genotyped and analyzed for 54 SNPs in the following genes: *TGFB2* (Transforming growth factor, beta 2), *HBB* (β-globin), *CD36* (Thrombospondin receptor), *HMOX1* (*Heme oxygenase 1*) and *ICAM 1* (*Intercellular adhesion molecule 1*, *CD54*). A complete list of genotyped SNPs is available in Table S1.

When selecting SNPs to genotype we prioritized those that were previously associated with CM, preferentially confirmed by sequencing and those belonging to distinct LD blocks as judged from the HapMap database (http://www.hapmap.org). The SNP genotyping method used the Mass Array system to design multiplex reactions for PCR and iPlex primer extension (Sequenom) and the MALDI-TOF based Mass Array genotype platform (Sequenom). Genotyping quality control selected SNPs that yield correct genotypes according HapMap control samples, passed the Hardy-Weinberg equilibrium test (P>0.05) with a calling rate >90%.

### *HMOX1* gene specific expression by qRT-PCR

For gene-specific expression, total RNA was obtained from 500 µl of whole blood from 42 CM patients collected to RNAlater solution (to stabilize RNA) using the RiboPure-Blood Kit (Ambion). One microgram of total RNA was converted to cDNA (Transcriptor High Fidelity cDNA Synthesis Kit, Roche) and *HMOX1* expression was quantified using TaqMan Gene Expression Assay Hs00157965_m1 from ABI with TaqMan Gene Expression master mix. The qRT-PCR was performed according to the manufacturer instructions on an ABI Prism 7900HT system. Relative quantification of *HMOX1* specific mRNA was obtained after normalization for *HPRT1* mRNA expression measured in the same PCR reaction.

### Data analysis

Data analysis followed a step-wise strategy. Firstly, case-control association analysis was performed for individual SNPs. Genotypic association of SNPs with susceptibility to malaria was analyzed using logistic regression models implemented in the SNPassoc package [54] for the R statistical software (version 2.7.0). An uncorrected significance level of the likelihood ratio test (P-value <0.05) was considered as suggestive evidence for association. Secondly, linkage disequilibrium (LD) maps were constructed exclusively for genes that demonstrated multiple SNPs associated to CM only when comparing CM cases to SnC controls. This analysis only pursued for genes that showed multiple polymorphisms associated to CM, exclusively when comparing CM patients to SnC patients. Pairwise LD was estimated using the correlation coefficient (r^2^) and the LD map was visualized using the snp.plotter R package [55] and was used to identify LD blocks within gene regions. In a third step, we used the *gene counting* method available in the R package GAP to reconstruct haplotypes composed with multiple associated SNPs within an LD block [56]. We intended to determine whether particular genotypic combinations of the CM-associated SNPs could confer a single joint specific effect on the risk of developing CM. Association between haplotypes and susceptibility to malaria was also evaluated with logistic regression analysis, using the same package that calculates global and haplotype-specific score statistics. Significant global score statistics (GSS) indicate that haplotype-specific scores (hap-score) are valid. A significant positive hap-score means that the haplotype confers susceptibility to the trait and a negative hap-score relates to protection [57]. For each haplotype, empirical P-values were calculated by permutation tests using the Monte-Carlo method to generate 1000 data simulations.

Finally, a family-based test using the TRANSMIT software [58] was performed for *HbS* and for haplotypes significantly associated to CM. Due to difficulties in collecting both parents, we took the conservative option of restricting the analysis to mother-descendent pairs performing the TDT analysis in children and their mothers.

Haplotypic quantitative trait association analysis for *HMOX1* gene expression levels was analyzed by the logistic regression model implemented in the Plink Package (version number 1.06) which calculates P values using empirical significance (http://pngu.mgh.harvard.edu/purcell/plink/) [59].

## Supporting Information

Table S1 Genotyped single nucleotide polymorphisms.
(0.08 MB DOC)

Table S2 Allelic distribution of the HMOX1 GT repeat in distinct patient groups and uninfected controls.
(0.04 MB DOC)

Table S3 Cerebral malaria association tests for single-nucleotide polymorphisms (SNPs) in the HMOX1, CD36 and ICAM1 genes.
(0.07 MB DOC)

Figure S1 Relative frequency distribution of the HMOX1 repeat alleles in distinct malaria phenotypes.
(0.06 MB DOC)
