# Supplementary material for: Transforming Growth Factor Beta 2 and Heme Oxygenase 1 Genes Are Risk Factors for the Cerebral Malaria Syndrome in Angolan Children
Source: PLoS One. 2010 Jun 16;5(6):e11141. doi: 10.1371/journal.pone.0011141 (PMC2886838; doi:10.1371/journal.pone.0011141)
Supplement: Table S1 — Genotyped single nucleotide polymorphisms. (0.08 MB DOC) [file pone.0011141.s001.doc]

**Sambo et al. 2010 (Supplementary data)**

**Table S1. Genotyped Single nucleotide Polymorphisms.**

| **Gene** | **Chromosome** | **SNP** | **Position (bp)** |
| --- | --- | --- | --- |
| TGFB2 | 1 | rs1418554 | 216589886 |
|  |  | rs1418555 | 216590273 |
|  |  | rs1417488 | 216590353 |
|  |  | rs4846476 | 216592851 |
|  |  | rs12097734 | 216607090 |
|  |  | rs4625350 | 216609934 |
|  |  | rs6691619 | 216615038 |
|  |  | rs6604607 | 216618910 |
|  |  | rs3892225 | 216619920 |
|  |  | rs2799086 | 216622825 |
|  |  | rs947712 | 216631504 |
|  |  | rs2799090 | 216635452 |
|  |  | rs6703224 | 216641412 |
|  |  | rs1317681 | 216641825 |
|  |  | rs1891467 | 216646608 |
|  |  | rs2796821 | 216652444 |
|  |  | rs12405805 | 216658738 |
|  |  | rs1934852 | 216661543 |
|  |  | rs6657275 | 216663084 |
|  |  | rs6671370 | 216663667 |
|  |  | rs1342586 | 216664482 |
|  |  | rs4846478 | 216664951 |
|  |  | rs6684205 | 216676325 |
|  |  | rs1418553 | 216676877 |
|  |  | rs900 | 216681528 |
|  |  | rs1473527 | 216688942 |
| CD36 | 7 | rs1194182 | 80069440 |
|  |  | rs1049654 | 80113391 |
|  |  | rs3211808 | 80114427 |
|  |  | rs3211810 | 80114953 |
|  |  | rs3211811 | 80115254 |
|  |  | rs3211820 | 80116401 |
|  |  | rs3211821 | 80116499 |
|  |  | rs3211849 | 80121259 |
|  |  | rs1358337 | 80126321 |
|  |  | rs3211909 | 80132051 |
|  |  | rs3211958 | 80142008 |
|  |  | rs7755 | 80144207 |
| HBB | 11 | rs334 | 5204808 |

**Continued.**

Table S1. (Continuation)

| **Gene** | **Chromosome** | **SNP** | **Position (bp)** |
| --- | --- | --- | --- |
| ICAM1 | 19 | rs5490 | 10242827 |
|  |  | rs5030351 | 10246417 |
|  |  | rs5491 | 10246540 |
|  |  | rs281432 | 10251658 |
| HMOX1 | 22 | rs2071746 | 34106672 |
|  |  | rs2071748 | 34107618 |
|  |  | rs17878600 | 34104876 |
|  |  | rs8139532 | 34109568 |
|  |  | rs7285877 | 34111214 |
|  |  | rs11912889 | 34113617 |
|  |  | rs9622194 | 34116963 |
|  |  | rs2285112 | 34119263 |
|  |  | rs5999817 | 34121108 |
|  |  | rs17883419 | 34121971 |
