# Supplementary material for: Transforming Growth Factor Beta 2 and Heme Oxygenase 1 Genes Are Risk Factors for the Cerebral Malaria Syndrome in Angolan Children
Source: PLoS One. 2010 Jun 16;5(6):e11141. doi: 10.1371/journal.pone.0011141 (PMC2886838; doi:10.1371/journal.pone.0011141)
Supplement: Table S2 — Allelic distribution of the HMOX1 GT repeat in distinct patient groups and uninfected controls. (0.04 MB DOC) [file pone.0011141.s002.doc]

**Sambo et al. 2010 (Supplementary data)**

**Table S2. Allelic distribution of the *HMOX1* GT repeat in distinct patient groups and uninfected controls**.

| Phenotype (n) | Number of GT repeats  N(%) | | | |
| --- | --- | --- | --- | --- |
|  | <24 | 24-28 | >28-34 | >34 |
| CM (92) | 46 (25.0) | 29 (15.8) | 63 (34.2) | 46 (25.0) |
| SnC (139) | 50 (18.0) | 48 (17.3) | 90 (32.4) | 90 (32.4) |
| UM (137) | 40 (14.6) | 45 (16.4) | 102(37.2) | 87 (31.8) |
| UIF (211) | 62 (14.7) | 91 (21.6) | 132 (31.3) | 137 (32.5) |

Abbreviations: CM, cerebral malaria; SnC, severe no cerebral malaria; UM, uncomplicated malaria; UIF, uninfected**.** Pearson's Chi-squared test: CM versus SnC (P= 1.7x10^-1^), CM versus UM (P= 3.8x10^-2^), CM versus UIF (P= 5.7x10^-3^).
