# Supplementary material for: Transforming Growth Factor Beta 2 and Heme Oxygenase 1 Genes Are Risk Factors for the Cerebral Malaria Syndrome in Angolan Children
Source: PLoS One. 2010 Jun 16;5(6):e11141. doi: 10.1371/journal.pone.0011141 (PMC2886838; doi:10.1371/journal.pone.0011141)
Supplement: Table S3 — Cerebral malaria association tests for single-nucleotide polymorphisms (SNPs) in the HMOX1, CD36 and ICAM1 genes. (0.07 MB DOC) [file pone.0011141.s003.doc]

**Sambo et al. 2010 (Supplementary data)**

Table S3. Cerebral malaria association tests for Single-Nucleotide Polymorphisms (SNPs) in the HMOX1, CD36 and ICAM1 Genes.

| **GENE** | **dbSNP**  **(RA)** | **RA frequency (%)** | | | | **Odds Ratio (CI)** | | |
| --- | --- | --- | --- | --- | --- | --- | --- | --- |
|  |  | **CM**  **(2n=260)** | **SnC**  **(2n=316)** | **UM**  **(2n=284)** | **UIF**  **(2n=638)** | **CM/SnC** | **CM/UM** | **CM/UIF** |
| CD36 | rs1049654  (C) | 45.3 | 39.7 | 40.0 | 39.0 | 1.24 (0.89-1.72) | 1.24 (0.89- 1.74) | 1.26 (0.95-1.68) |
|  | rs3211808  (T) | 86.8 | 87.8 | 88.0 | 85.7 | 1.09 (0.67-1.75) | 1.10 (0.67-1.80) | 1.08 (0.72- 1.61) |
|  | rs3211810  (T) | 85.4 | 85.0 | 85.0 | 85.5 | 1.03 (0.64-1.67) | 1.03 (0.64-1.69) | 1.01 (0.66-1.52) |
|  | rs3211811  (T) | 12.2 | 10.6 | 11.3 | 12.8 | 1.18 (0.69-2.03) | 1.09 (0.64-1.83) | 1.05 (0.68-1.64) |
|  | rs3211820  (C) | 77.9 | 71.6 | 71.7 | 73.8 | 1.39 (0.93-2.04) | 1.37 (0.90-2.08) | 1.25 (0.56-1.14) |
|  | rs3211849  (A) | 37.3 | 36.1 | 36.6 | 36.6 | 1.05 (0.75-1.49) | 1.03 (0.73- 1.44) | 1.03 (0.76-1.39) |
|  | rs3211909  (C) | 29.7 | 30.9 | 32.3 | 28.6 | 1.05 (0.74-1.52) | 1.14 (0.76-1.69) | 1.06 (0.75-1.48) |
|  | rs3211958  (A) | 88.9 | 88.2 | 88.3 | 87.7 | 1.06 (0.64-1.79) | 1.06 (0.61-1.89) | 1.00 (0.61-1.64) |
|  | rs7755  (G) | 84.9 | 82.7 | 83.3 | 82.1 | 1.18 (0.75-1.82) | 1.12 (0.70- 1.78) | 1.21 (0.82-1.79) |
| ICAM 1 | rs281432  (G) | 77.0 | 73.0 | 77.2 | 75.0 | 1.28 (0.84-1.96) | 1.01 (0.68-1.51) | 1.1 (0.79-1.59) |
|  | rs5030351  (C) | 76.2 | 76.8 | 70.8 | 74.7 | 1.04 (0.70-1.52) | 1.33 (0.90-1.96) | 1.09 (0.77- 1.54) |
|  | rs5490  (A) | 71.4 | 76.0 | 75.9 | 64.4 | 1.35 (0.65-2.77) | 1.34 (0.68-2.63) | 1.67 (0.75-1.49) |
|  | rs5491  (A) | 75.0 | 75.8 | 69.9 | 74.0 | 1.05 (0.71-1.53) | 1.30 (0.88-1.92) | 1.05 (0.75-1.49) |
| HMOX1 | rs17878600 (C) | 64.0 | 60.0 | 61.0 | 61.0 | 1.20 (0.85-1.72) | 1.21 (0.74-2.0) | 1.18 (0.77-1.78) |
|  | rs2071746 (T) | 76.0 | 78.0 | 71.0 | 72.0 | 1.08 (0.67-1.75) | 1.41 (0.86-2.72) | 1.22 (0.87-1.72) |
|  | rs9622194 (G) | 56.3 | 50.7 | 55.7 | 54.0 | 1.05 (0.61-1.85) | 1.20 (0.72-2.0) | 1.10 (0.82-1.47) |
|  | rs2285112 (G) | 76.7 | 79.5 | 70.9 | 74.6 | 1.05 (0.65-1.70) | 1.54 (0.95-2.5) | 1.19 (0.79-1.82) |
|  | rs5999817 (T) | 12.0 | 11.5 | 12.2 | 11.5 | 1.14 (0.65-2.00) | 1.01 (0.58 -1.79) | 1.10 (0.67-1.79) |
|  | rs17883419  (C) | 88.0 | 88.7 | 84.4 | 86.3 | 1.04 (0.59-1.83) | 1.09 (0.15-7.89) | 1.19 (0.73-1.92) |

Abbreviations: RA, reference allele; CM, cerebral malaria; SnC, severe no cerebral malaria; UM, uncomplicated malaria; UIF, uninfected. Allele frequencies refer to the reference alleles. P-values (uncorrected) and Odds Ratios refer to genotypic analysis and were obtained by logistic regression analysis.
