# Supplementary material for: Transforming Growth Factor Beta 2 and Heme Oxygenase 1 Genes Are Risk Factors for the Cerebral Malaria Syndrome in Angolan Children
Source: PLoS One. 2010 Jun 16;5(6):e11141. doi: 10.1371/journal.pone.0011141 (PMC2886838; doi:10.1371/journal.pone.0011141)
Supplement: Figure S1 — Relative frequency distribution of the HMOX1 repeat alleles in distinct malaria phenotypes. (0.06 MB DOC) [file pone.0011141.s004.doc]

**Sambo et al. 2010 (Supplementary data)**


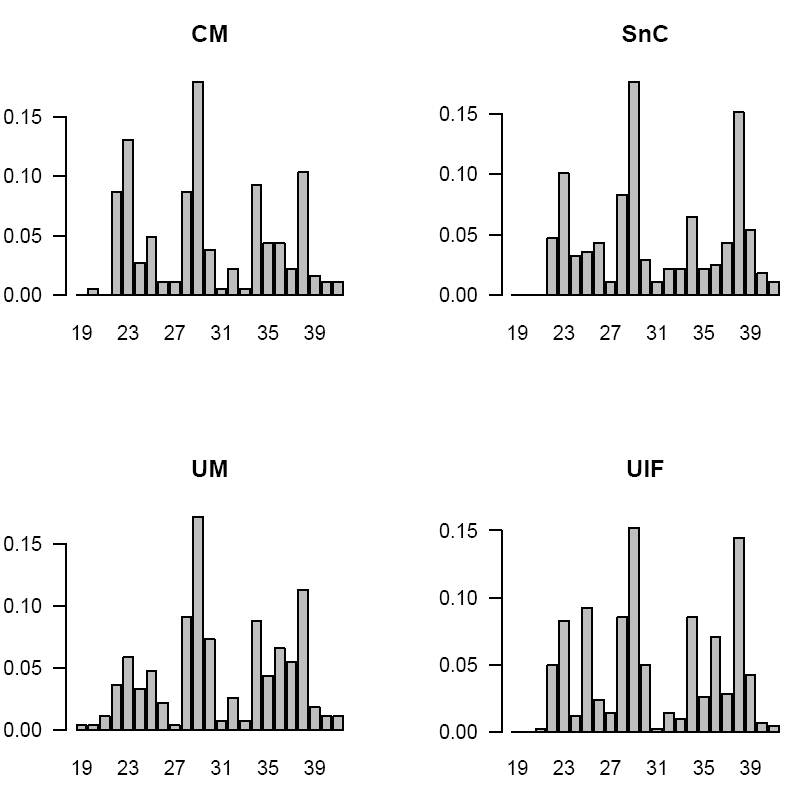


**Figure S1. Relative frequency distribution of the *HMOX1* repeat alleles in distinct malaria phenotypes**. Y-axis represents relative frequency and the X-axis the allele repeat number. The 5'-flanking region of the HO-1 gene containing a poly (GT)n repeat was amplified by the polymerase chain reaction (PCR) using a fluorescent-labeled sense primer (5'-FAM-AGAGCCTGCAGCTTCTCAGA-3') and an unlabeled antisense primer (5'-ACA AAGTCTGGCCATAGG AC-3'). The sizes of PCR products were analyzed using an internal size-standard (GeneScan ROX 350 size standard, Applied Biosystems, Foster City, CA), on a laser-based ABI Prism®3100 automated DNA capillary Sequencer (Applied Biosystems, Foster City, CA). Fragment length GT-repeat length attribution was done semi automatically using ABI Prism Software (Gene Scan Analysis Version 3.7 and Genotyper Software Version 3.7, both Applied Biosystems, Foster City, CA).
